# Editorial: The role of cancer associated fibroblast (CAF) in EMT/metastasis in malignancies of epithelial tissues

**DOI:** 10.3389/fcell.2024.1384150

**Published:** 2024-02-28

**Authors:** Biplab Giri, Tapasree Roy Sarkar

**Affiliations:** ^1^ Department of Physiology, University of Gour Banga, Malda, West Bengal, India; ^2^ Department of Biology, Texas A&M University, College Station, TX, United States

**Keywords:** cancer associated fibroblast (CAF), tumor microenvironment (TME), metastasis, breast cancer, oral squamous cell carcinoma (OSCC), extracellular vesicles (EVs), notch signaling in TME

The tumor microenvironment (TME) plays a crucial role in cancer due to its intricate interactions with the tumor. Within the TME, Cancer Associated Fibroblasts (CAFs) are a diverse group of cells that have a significant impact on tumor growth, metastasis, treatment resistance, and relapse. In the dynamic field of oncology, TME has become a pivotal factor, with a particular focus here on CAFs. This highlights the urgent need to merge the latest scientific discoveries to advance cancer research and develop precise therapies. Our Research Topic includes four informative articles that collectively delve into the multifaceted impact of CAFs on cancer progression, offering a subtle perspective that could inspire novel therapeutic approaches.

In the first article, “CAFs: The Chief Architect in TME,” the intricacies and variations of CAFs are explored, shedding light on their imperative role in driving tumor development by initiating stemness, spreading to other parts of the body, and resistance to treatment. These functions are facilitated by extracellular matrix modification, immunomodulation, and altering metabolism. It is essential to recognize the diverse CAF populations in order to effectively address obstacles in cancer treatment. One of the most pressing challenges is tackling therapy resistance which can significantly impact patient outcomes. However, targeting CAFs for treatment proves to be a formidable roadblock as they do not possess distinct markers (like αSMA or FAP), making it challenging to directly target them without causing harm to healthy cells. This highlights the significance of delving deeper into the multifaceted roles played by CAFs in driving cancer progression and developing more successful strategies for treatment. Here, the current understanding of the origin and heterogeneity of CAFs, their role in tumor progression, and altering the tumor response to therapeutic agents specifically in breast cancer has been discussed, which may be useful in developing CAF-mediated therapeutic strategies against cancer (Sarkar et al.).

In the subsequent article, “Extracellular Vesicles and Pro-inflammatory CAF Activation in Oral Cancer,” an intriguing new angle is investigated regarding the activation of CAFs in oral squamous cell carcinoma (OSCC). OSCC cells release extracellular vesicles (EVs) which stimulate the activity of CAFs, revealing a unique method of communication between tumors and the surrounding tissue. Delving into this phenomenon, this research has discovered that OSCC-derived EVs trigger a specialized pro-inflammatory CAF state (known as iCAF) marked by increased expression of inflammatory cytokines, IL-8 and CXCL5, and genes involved in hypoxia. The activated CAFs induced by EVs show distinct gene expression profiles compared to those activated by TGFβ, indicating varied roles in tumor progression and immune function. Of particular note, these EV-activated CAFs may play a role in creating an immunosuppressive environment within the TME. This highlights the role of EV-based communication in the development of OSCC, the intricate nature of CAF diversity regulated by various stimuli in the TME, and proposes the possibility of targeting specific CAF subtypes, potentially through influencing EV contents, as a promising approach for treating OSCC (Arebro et al.).

The article titled “CD105 Expression in CAFs: A Biomarker for Bone Metastasis in Breast Cancer” presents innovative advancements in identifying prognostic markers for bone metastasis in patients with early invasive ductal breast cancer. This research delves into the potential impact of CD105 expression in CAFs on early-stage breast cancer patients. Analysis of 342 invasive ductal breast carcinomas reveals a significant correlation between elevated CD105 levels in CAFs and the higher likelihood of bone metastasis and a decrease in overall survival. This suggests that CD105+ CAFs play a crucial role in the aggressive nature and potential for metastasis of the tumor (Giorello et al.).

In the fourth article, titled “The Role of NOTCH Signaling in CAF-mediated Metastasis,” the intricate molecular workings of CAFs are explored, with a specific emphasis on the Notch signaling pathway. Even though the oncogenic properties of Notch in cancer development are well-established, its functions within the TME and its interaction with CAFs are still not fully understood. The understanding of the diverse nature of CAFs and the complex array of Notch signaling in the TME could potentially lead to novel therapeutic approaches that target both the primary tumor and its spread by reprogramming the tumor stroma (Ghosh and Mitra).

In conclusion, these articles serve as a collective cue of the intricate dynamics present within the TME, with a particular emphasis on the significant role of CAFs in driving cancer progression. They call for a shift in our conventional approach to cancer treatment—one that focuses on targeting cancer cells and also recognizes the importance of disrupting the intricate network of interactions within the TME, particularly involving CAFs. Furthermore, these insightful studies deepen our understanding of cancer biology and also provide a solid foundation for developing more targeted and efficient treatments. The ongoing exploration of the TME continues to highlight the pivotal role of CAFs, serving as a guiding light toward innovative strategies to combat this complex and ever-evolving disease.

